# Temporary Nerve Block at Selected Digits Revealed Hand Motor Deficits in Grasping Tasks

**DOI:** 10.3389/fnhum.2016.00596

**Published:** 2016-11-25

**Authors:** Aude Carteron, Kerry McPartlan, Christina Gioeli, Emily Reid, Matt Turturro, Barry Hahn, Cynthia Benson, Wei Zhang

**Affiliations:** ^1^Department of Physical Therapy, College of Staten Island, City University of New YorkStaten Island, NY, USA; ^2^Emergency Medicine, Staten Island University HospitalStaten Island, NY, USA; ^3^Ph.D. Program in Biology, Graduate School and University Center, City University of New YorkNew York, NY, USA

**Keywords:** hand, anesthesia, anticipatory control, force, moment, motor coordination, motor synergy

## Abstract

Peripheral sensory feedback plays a crucial role in ensuring correct motor execution throughout hand grasp control. Previous studies utilized local anesthesia to deprive somatosensory feedback in the digits or hand, observations included sensorimotor deficits at both corticospinal and peripheral levels. However, the questions of how the disturbed and intact sensory input integrate and interact with each other to assist the motor program execution, and whether the motor coordination based on motor output variability between affected and non-affected elements (e.g., digits) becomes interfered by the local sensory deficiency, have not been answered. The current study aims to investigate the effect of peripheral deafferentation through digital nerve blocks at selective digits on motor performance and motor coordination in grasp control. Our results suggested that the absence of somatosensory information induced motor deficits in hand grasp control, as evidenced by reduced maximal force production ability in both local and non-local digits, impairment of force and moment control during object lift and hold, and attenuated motor synergies in stabilizing task performance variables, namely the tangential force and moment of force. These findings implied that individual sensory input is shared across all the digits and the disturbed signal from local sensory channel(s) has a more comprehensive impact on the process of the motor output execution in the sensorimotor integration process. Additionally, a feedback control mechanism with a sensation-based component resides in the formation process for the motor covariation structure.

## Introduction

Peripheral sensory feedback plays a crucial role in the correct execution of a voluntary movement (Nowak et al., 2003, 2004; Weiss et al., 2011; Stone and Gonzalez, 2015). A continuously updated cyclic sensorimotor integration process is required to successfully complete a motor task. Each cycle begins with peripheral pathways conveying sensory information, followed with the central nervous system integrating the sensory inputs to assist motor program execution (Abbruzzese and Berardelli, 2003). Thus, abnormalities in the peripheral afferent inputs, or in their central processing, may interfere with motor program execution, leading to motor function deficits (Rossini et al., 1996; Rossi et al., 1998; Nowak et al., 2003; Richardson et al., 2016). Additionally, those abnormalities partially contribute to the motor disorder under pathophysiological conditions (Hallett, 1995; Kaji et al., 1995; Fellows et al., 1998; Marchese et al., 2000; Quinn et al., 2001; Schwarz et al., 2001; Sens et al., 2013; Zhang and Santello, 2014). Despite the apparent importance of afferent inputs in the process of sensorimotor integration, its explicit role in voluntary movement control mechanism is not well understood.

To explore the contribution of somatosensory feedback in hand manipulation tasks, object grasping has been used as an effective model (Lederman and Klatzky, 1990, 2009; Stone and Gonzalez, 2014a,b). Previous studies revealed that absent or disturbed afferent inputs interfered with the sensorimotor integration process during steady or dynamic prehensile tasks. Local anesthesia procedures have been applied to individual digits (Witney et al., 2004) or to the lower median nerve (Li et al., 2004; Li and Nimbarte, 2006) to block peripheral somatosensory feedback (Gissen et al., 1982; Witney et al., 2004). These studies suggested that digital deafferentation introduced multiple motor deficits in force modulations. Force deficits include deteriorated maximal grip force production (Augurelle et al., 2003; Pavlova et al., 2015), excessively large grip force to lift or hold an object (Johansson and Westling, 1984; Johansson et al., 1992; Nowak et al., 2001; Monzée et al., 2003; Dun et al., 2007), delayed force development or force adaptation to perturbation (Johansson and Westling, 1984; Johansson et al., 1992; Jenmalm and Johansson, 1997; Monzée et al., 2003), disturbed coupling of normal and tangential forces (Nowak et al., 2001; Augurelle et al., 2003), and inaccurate moment of force production patterns (Monzée et al., 2003; Li and Li, 2013). Although none of these studies investigated the whole hand grasp control (i.e., only two-digit pinch or three-digit grasp was employed), their findings revealed an impaired sensorimotor integration process following somatosensory feedback deprivation at peripheral levels. At corticospinal levels, neurophysiological studies suggested that cutaneous feedback deprivation decreases the activation of motor neurons in several parts of the central nervous system (Rossini et al., 1996; Rossi et al., 1998), such as the dorsal horn of the spinal cord (Koerber and Brown, 1995), thalamus (Nicolelis et al., 1993; Rasmusson, 1996), and the cerebral cortex (Wall et al., 1986). However, most previous studies focused on the effect of local afferent abnormalities on adaptive peripheral behavioral and central processing changes, and none of them addressed the following questions: (1) how the disturbed and intact sensory input integrate and interact with each other to modulate the motor program execution; and (2) whether the motor coordination based on motor output variability presents between affected and non-affected elements (e.g., digits) and becomes interfered by the local sensory deficiency.

Some recent studies explored the sensorimotor integration process in carpal tunnel syndrome (CTS) patients (Zhang and Santello, 2014). Given reduced tactile sensation solely at a subset of digits (i.e., the thumb, index, middle and lateral half of the ring finger), CTS patients demonstrated excessive grip force (Zhang et al., 2011), decreased motor learning and adaptation (Zhang et al., 2012), lower force discrimination, and non-efficient torque production (Zhang et al., 2011). Most interestingly, the patients’ excessively large force was presented only when the non- or less-affected fingers were employed together with CTS-affected digits in the grip task (Zhang et al., 2013). These findings suggest a larger challenge for the CNS to integrate disturbed with intact peripheral sensory channels in a motor task adaptation. The purpose of the current study is to investigate the multi-digit motor performance and coordination within and between deafferent and intact digits during hand grasping control tasks. Digital nerve block was applied to temporarily remove the somatosensory feedback from a subset of digits, but not the entire hand. We expect that the absence of somatosensory input from the digits would induce motor deficits in both grasp force control and moment control, and would deteriorate the motor coordination between deafferent and intact digits.

## Materials and Methods

### Subjects

Nineteen (10 females and 9 males) right-handed young adults participated in the current study (age (mean ± SD): 23.32 ± 0.37 years old, height: 1.69 ± 0.02 m, weight: 72.35 ± 2.99 kg, hand width and length: 8.28 ± 0.17 cm and 18.11 ± 0.25 cm respectively). Subjects had no revealed history of neurological, musculoskeletal, vascular or metabolic disorders, impairment of their right upper limb, or allergy history for the anesthetic agent and preparation material. The study protocol was approved by the Institutional Review Board at the City University of New York and North Shore-Long Island Jewish Health system. All subjects were naïve to the purpose of the study and gave their written informed consent according to the Declaration of Helsinki.

### Apparatus

**Figure 1:**
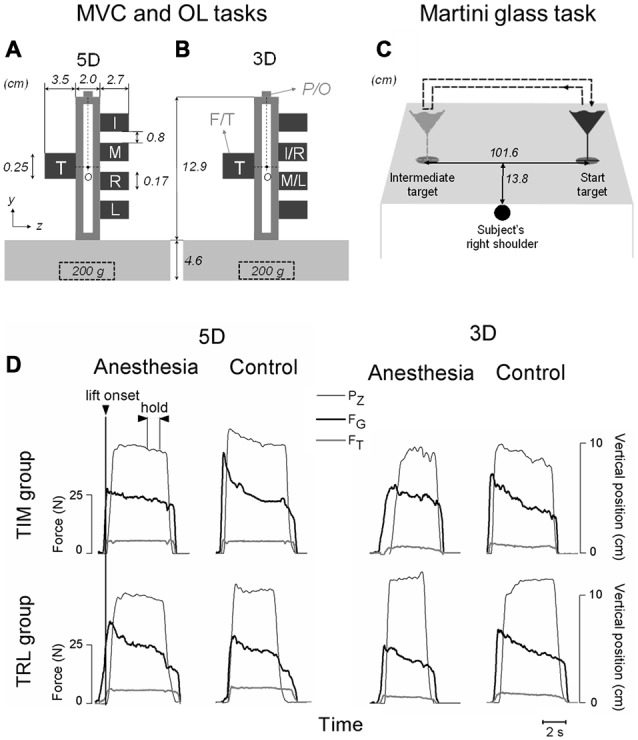
**Experimental setup and variables. (A,B)** Show the front view of the customized grip device utilized for maximal voluntary contraction (MVC) and object lifting (OL) tasks. Five force/torque (F/T) sensors are mounted on both sides of the device to measure forces and moment of forces exerted by individual digits involved in five-digit grip (5D; thumb, index, middle, ring and little fingers: T, I, M, R and L, respectively) in **(A)** or three-digit grip (3D; TIM or TRL) in **(B).** A mass of 200 g was inserted in the midpoint at the bottom of the grip device throughout the tasks. Note that all five sensors were mounted on the grip device to maintain a constant weight for both grip types, even though only the two intermediate sensors were used for I and M (or R and L) in 3D. A magnetic tracker position/orientation (P/O) attached on the top of the device was used to measure the object position and orientation during the manipulation. “O” denotes the point about which moments were computed (see “Materials and Methods” Section for more details). **(C)** Demonstrates an auxiliary task (Martini glass task) when a martini glass filled with water was to be lifted by using a five-digit grip, transported from a start target location to an intermediate target location, and transported back to the origin location. A dashed arrow line denotes the glass transportation trajectory. Subject’s right shoulder was aligned with the center between the two target locations. **(D)** Shows the time course of object vertical position (*P*_Z_), grip force (F_G_) and tangential force (F_T_) performed by one representative subject before (Control) and after digital nerve block procedure (Anesthesia) from either TIM or TRL group by using three- and five-digit grip types. All data traces are aligned with object lift onset (vertical line). Plotted data in each subpanel are from a subsequent trial in OL task. Experimental variables were analyzed and extracted at two defined task epochs: at object lift onset and during object hold.

An inverted T-shape customized grip device was applied in the current study to allow for comfortable grasp. Five force/torque (F/T) transducers (Nano-25 for the thumb and Nano-17 for four fingers, ATI Industrial Automation, Apex, NC, USA) were installed to measure local forces and torques in three dimensions at individual digits, i.e., thumb, index, middle, ring and little (T, I, M, R and L respectively; Figure 1A). Note that when only three digits were used in the grasp tasks (3D, see procedure below), the F/T sensor for the thumb and the two intermediate sensors on the fingers side were used instead (Figure 1B). All sensors were covered by 100-grit sandpaper to increase grip friction and prevent slipping. One electromagnetic tracking sensor (Polhemus Fastrak, Colchester, VT, USA; 0.075 mm and 0.05 u resolution) was attached on the top of the device to measure its position/orientation (P/O). The grip device was loaded with a 200 g mass in the center at the bottom to centralize its mass distribution and achieve adequate yet comfortable weight (665 g in total) for the lifting task. Note that both the mass and mass distribution remained the same throughout the experiment. Dimensions of the device are indicated in Figures 1A,B. Five 12-bit A/D converted boards recorded the F/T data at a sampling frequency of 1 kHz, and P/O data were recorded at 80 Hz. All the measurements were acquired, displayed and stored through a customized program in LabVIEW 2010 (National Instruments).

### Experimental Procedures

All subjects completed the current study in two sessions with a 2-week interval: the first session will be referred to as *control* session while the second one, where a standard digital nerve block procedure was conducted, will be referred to as *anesthesia* session. Subjects were assigned randomly into one of two groups according to the digits to be anesthetized during the anesthesia session:* TIM* group (5F, 5M) and* TRL* group (5F, 4M), with digital anesthesia applied for thumb, index and middle and for thumb, ring and little fingers separately.

In both control and anesthesia sessions, each subject was instructed to complete two primary experimental tasks, including a maximal voluntary contraction (MVC) task and an object lifting (OL) task. Additionally, subjects completed an auxiliary experimental task, i.e., martini glass task. During the experiment, subjects sat comfortably 10–15 cm away from a test table and faced a grip object (a grip device or a martini glass according to the task). The grip object was placed within arm’s reach to allow comfortable reaching with minimal trunk movement. The grip device was not fixed on the top of the desk throughout the experiment. Before the experiment, subjects were asked to rest their right hand on the table without touching the grip object. For both MVC and OL tasks, the subject’s right shoulder was aligned with the grip device.

In the MVC task, subjects’ peak grip force were measured to evaluate their maximal force production ability with the hand in prehensile position. After a verbal “go” signal, subjects were instructed to reach the grip device from a hand rest position, contact the tip of all task-involved digits on corresponding individual F/T sensors, squeeze as strong as possible within 8 s without lifting the device from the top of the desk, and relax after their peak maximal force was reached. Subjects were provided with an online feedback by displaying their total grip force-time profile on a computer monitor. Displayed force was always scaled to subject’s maximal force amplitude to ensure visual feedback for full range of force. MVC task consisted of three grip conditions according to the digits combination instructed to be used, namely: (1) 5D: all five digits; (2) 3D_ane: all three digits selected to be anesthetized in the anesthesia session (i.e., T, I and M for TIM group, and T, R and L for TRL group); and (3) 3D_mix: a three digit combination of the thumb and two non-anesthetized fingers (i.e., T, R and L for TIM group, and T, I and M for TRL group). Subjects completed two trials for each condition resulting in six trials in total in MVC task.

In OL task, subjects were instructed to reach the grip device after a verbal “go” signal, contact task-involved digits on the surface of individual sensors, lift the grip device ~10 cm above the table at a self-selected pace, hold it in the air for 4 s, and replace it on the table. Two different *grip types* were used in OL task: a five-digit and three-digit grip (5D and 3D, respectively). Note that the three digits used in the 3D grip were all anesthetized digits (i.e., T, I and M for TIM group, and T, R and L for TRL group). After three practice trials, subjects performed 25 trials with each grip type, resulting in 56 trials in total in OL task. The instruction “lift and hold the object as vertical as possible” was consistently repeated throughout the OL task for all the subjects.

The auxiliary experimental task, martini glass task (Figure 1C), was designed to mimic a daily-life hand activity with the addition of interference to the sensorimotor integration process. Before the martini glass task, subjects rested their right hand on the desk, which was aligned with their right shoulder and the midpoint between “Start target” and “Intermediate target”, the original and intermediate position of the martini glass respectively (Figure 1C). After a verbal “go” signal, subjects reached their right hand to a standard martini glass filled with water (436 g including 352 g of water) located at “Start target”, gripped it by placing their five digits on the glass stem only, transported it to “Intermediate target” without releasing nor changing digit placement, and replaced it back at “Start target”. Subjects were required to complete the martini glass transportation as quick as possible while minimizing water spill. Each subject performed the martini glass task for three successive trials. The time duration of each trial was recorded and the cumulated mass of water spilled over the three trials was calculated after weighting the martini glass with the water remaining.

At least 30 s between-trials and 5 min between-tasks pauses were observed during the experiment. Subjects were given necessary rest time to prevent fatigue and additional rest time upon individual request.

### Digital Nerve Block at Selective Digits

During the anesthesia session, a standard digital nerve block procedure was conducted by the same licensed physician (Staten Island University Hospital, New York, NY, USA) for all the subjects prior to the experimental tasks described above. Local anesthetic (1% lidocaine and 0.5% bupivicaine) injections were administered at digital nerves gradually and incrementally in the web space so that sensory nerve fibers were blocked including type C, type A delta, gamma and beta (pain, temperature, postural, touch and pressure), while motor nerve fibers (type A alpha) remained intact (Gissen et al., 1982)^1^. The physician determined the dosage of medication according to individual subject body weight. Additional dosage was injected if residual tactile sensations remained after initial injection, and up to three injections were conducted for the same digit per subject. Participants whose tactile sensation was not successfully blocked after receiving three injections were excluded from the study. A microfilament toolkit was used for all digits after each digital nerve block procedure and throughout the experiment to ensure the blockage of tactile sensation at three anesthetized digits but not at two un-anesthetized digits.

### Data Processing

Data analyses were performed with MATLAB 8.1.0 (MathWorks), Excel 14.0 (Microsoft) and SPSS 20 (IBM). Data from one subject (female in TIM group) was determined as an outlier, i.e., out of range of mean ± 3SD, and was therefore omitted from our data analysis and results. Figure 1D shows time courses of kinetic and kinematic data from representative subjects in TIM and TRL groups before and after their digital nerve block procedure (Control and Anesthesia respectively) by using three- or five-digit grip types.

For convenience, virtual finger (VF), a hypothetical entity that produces a mechanical output equivalent to the individual fingers combined (Arbib et al., 1985; Baud-Bovy and Soechting, 2001; Zhang et al., 2009), is used to denote all task-involved digits except the thumb. In the current study, we defined two substitutes: VF*a* to designate the set of two fingers that received the injection during the anesthesia visit (i.e., I and M for TIM subject group; R and L for TRL subject group), and VF*na* to designate the set of two non-anesthetized fingers on the VF side (i.e., R and L for TIM subject group; I and M for TRL subject group). In this scenario, different grip condition could be viewed as a combination of T, VF*a* and VF*na*: 5D involved T, VF*a* and VF*na*, whereas 3D grip conditions involved T and VF*a* or VF*na*.

In OL task, kinematics and kinetics data were temporally aligned offline by re-sampling position and orientation data through linear interpolation to match the force data sampling frequency. Experimental variables were extracted at two defined task epochs: (1) at object *lift onset*; and (2) during object *hold*. Object lift onset reflects anticipatory control behavior based on previous trials, whereas object hold provides an insight into motor adaptation resulting from the integration of sensory feedback acquired following lift onset (Zhang et al., 2010, 2011, 2012, 2013). These task epochs were determined for each individual trial based on the smoothed position data. Briefly, object lift onset was identified as the time when the derivative of the object’s vertical position (Pz) crossed a threshold (signal baseline mean + 3 SD) for more than 200 ms. The end of object hold was determined as the time during object release when the derivative of Pz dropped below 3% of its baseline at hold. As force transients occur at the beginning and by the end of the object hold period, experimental variables related to object hold were analyzed by averaging over the 1.2 s period of the steady portion 0.5 s prior to the end of object hold (Figure 1D).

All the mechanical variables of interest were in the frontal plane (Figures 1A,B). Experimental kinetics variables in MVC and OL tasks were processed based upon individual digit kinetics data, including: (1) digit *normal force* (Fn), which is the force component perpendicular to the grip surface used to grip on the object; (2) digit *tangential force* (Ft), which is the force component parallel to the grip surface to lift the object; and (3) digit *center of pressure* (CoP), which is the resultant force application point of each digit on the grip surface. We analyzed experimental variables as follows:

(1) *Grip force* (F_G_) was defined as the sum of Fn performed by all involved individual digits on the grip object.
(2) *Tangential force* (F_T_) was defined as the sum of Ft performed by all involved individual digits on the grip object.

### MVC Task

(3) *Maximal grip force* (Max_F_G_) denotes the peak value of F_G_ over each trial in MVC task. Furthermore, a larger Max_F_G_ was selected from the two collected trials for each subject per digit condition.
(4) Digit *force contribution* in MVC task denotes the Fn produced by one, or a combination of digits when all involved digits reached Max_F_G_.

### OL Task

(5) *Within-trial grip force adaptation* (ΔF_G_) was calculated as the difference between F_G_ at the object lift onset and F_G_ averaged during object hold. Our previous findings suggested a non-zero ΔF_G_ indicating a force adaptation resulting from an erroneous anticipatory control mechanism (Zhang et al., 2011).
(6) *Object roll* was defined as the absolute angle of deviation of the grip device from the vertical axis in the frontal plane. Maintaining the object orientation was required in OL task, and thus the *peak object roll* during object lift and averaged object roll during hold were used to determine subjects’ performance as consequences of anticipatory force, moment control and the following adaptation respectively.
(7) *Moment of forces*. Individual digit moment of force (M*i*) was calculated as the sum of the moment produced by Fn and the moment produced by Ft with respect to the center point of grip device “O” in the frontal plane (Figures 1A,B). Note that the *total moment of forces* (M_TOT_) applied to the device by all the involved digits should equal to zero to minimize object tilt in OL task.
(8) *A Synergy* index. We investigated how the digits coordinate with each other and quantified the multi-digit synergies based on a variance analysis (Latash et al., 2002; Zhang et al., 2009; SKM et al., 2012). An index (ΔV) of synergy was calculated as the normalized difference between the sum of the across-trial variances of elemental variables such as individual digit mechanical outputs and across-trial variance of the performance variable, i.e., the resultant output of the specified digits (Equation 1). In order to determine the multi-digit coordination at different hierarchies (Zhang et al., 2009), the synergy index was calculated at four levels: (a) *hand individual digit level* (*Hand_d*) involving T, I, M, R and L in 5D, and T, I, M or T, R, L in 3D; (b) *thumb-virtual finger level* (*T-VF*) involving T and VF in both 5D and 3D; (c) *VF individual finger level* (*VF_f*) involving I, M, R and L in 5D, and I, M or R, L in 3D; and (d) *VF anesthesia and non-anesthesia fingers level* (*VFa-VFna*) involving I, M and R, L in 5D *only*). At each hierarchical level, the multi-digit synergy was quantified with Equation 1 for three performance variables: normal force (ΔV_F_N_), tangential force (ΔV_F_T_) or resultant moment of forces (ΔV_M) of specified digits.

(1) $$\Delta V\_P=\frac{\sum_{i}Var(E_{i})-Var\sum_{i}E_{i}}{\sum_{i}Var(E_{i})}$$

In Equation 1 *E* denotes the elemental variable (i.e., individual digit’s Fn_,_ Ft or M). *P* denotes the performance variable studied (i.e., overall digits’ Fn_,_ Ft or M), which can be calculated as ∑_*i*_ *E*_i_. I denotes specified digits according to hierarchical levels. *Var* is the variance calculated across all 27 trials at two time epochs defined earlier (object lift onset and hold), for a given condition and grip type with first trial excluded. An emergence of a synergy stabilizing the resultant performance variable can be implied by positive values of ΔV (Δ*V* > 0), which indicate negative co-variation (error compensation, Latash et al., 1998; Zhang et al., 2009) among elemental variables from trial to trial. Inversely, a negative ΔV indicates an absence of multi-element synergy for the considered performance variable and level. The normalization by ∑_*i*_ *Var*(*E*_i_) allows comparison across subjects and conditions.

### Martini Glass Task

(9) *Total water loss*. The overall mass of water spilled over the three trials in martini glass task was evaluated to assess the overall task performance.
(10) *Time spent*. The duration of glass transportation of each trial was recorded to assess the task temporal performance.

### Statistical Analysis

Multi-factor analysis of variance (ANOVA) with repeated measures were performed for experimental variables described above with the within-subject factors of *Session* (Anesthesia, Control), *Grip type* (5D, 3D_ane, 3D_mix in MVC task; 5D and 3D in OL task), Digit units (T, VFa, VFna), *Phase* (Lift onset, Hold), *Hierarchical level* (*Hand_d, T-VF, VF_f, VFa-VFna*), and *Trial* (1, 2, 3), and with the between-subject factor of *Group* (TIM group, TRL group).

In the MVC task, we performed a 3-way ANOVA with repeated measures on the Max_F_G_ with the factors of *Session, Grip type* and* Group*. Furthermore, the individual digit force contribution to the Max_F_G_ was evaluated for each Grip type by separate 3-way ANOVAs with repeated measures with factors of *Session, Digit units* and *Group*.

In the Lifting task, a preliminary test involving the factor of Trial (levels: 1, 2, …, 28) was performed for each individual experimental variable. Given that the first trial was the only trial found to be significantly different from the others, this trial of learning was omitted, and an average of data or variance of data was calculated based on subsequent trials (2–28) in further analysis. To investigate the subjects’ performance with and without anesthetized digits, we performed a 3-way ANOVA with repeated measures on ΔF_G_ with factors of *Session, Grip type* and* Group*. In the aforementioned tests there was no main effect of *Group* nor interaction involving this factor, it was therefore removed from further statistical tests. Additionally, we performed a 3-way ANOVA with repeated measures on object roll with factors of *Session, Grip type and Phase*.

Given that the maximal value of synergy indices was limited by +1, we applied Ficher’s *z*-transformation to positive indices values before performing the statistics (Zhang et al., 2009) as follows: ΔV = 0.5 (ln(1 + ΔV)-ln(1 − ΔV)). One-Sample *T*-tests were used to define whether each synergy index (ΔV_F_N_, Δ*V*_F_T_ and ΔV_M) was different from zero. To determine the multi-digit coordination among anesthetized digits and/or non-anesthetized digits, we performed separate 3-way ANOVAs with repeated measures for each synergy index at each hierarchical level with the factors of *Session, Grip type* and* Phase*. In addition, to examine the multi-digit coordination at different hierarchical control levels, we performed separate 3-way ANOVAs with repeated measures with factors of *Session, Phase* and *Hierarchical level* for each synergy index in 5D and 3D grip type individually, since *Hierarchical level* in 5D has four levels yet in 3D has three levels.

In the martini glass task, to investigate subjects’ performance in a natural task with or without anesthetized digits, we performed a 2-way ANOVA with repeated measures on the total water loss with the factors of *Session* and *Group*, and a 3-way ANOVA with repeated measures on the time spent with *Session, Trial* and* Group*.

When the assumption of sphericity was violated, Greenhouse-Geisser correction of degrees of freedom was used and thus the adjusted *P* values were reported. *Post hoc* tests for pairwise comparisons were performed with Bonferroni adjustments when appropriate. The level of significance used was *P* < 0.05. All reported values are averages across subjects ± standard error of the mean.

## Results

All subjects in both anesthesia and control sessions were able to complete the experimental tasks as instructed, without dropping the device or showing fatigue during the experiment.

### MVC Task

**Figure 2:**
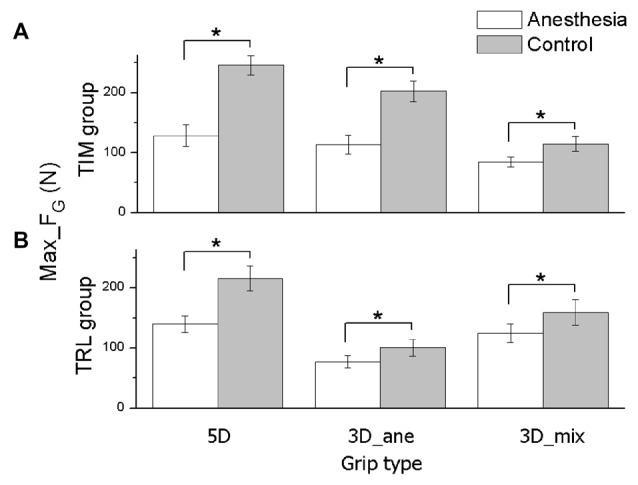
**Maximal grip force in MVC task.** The maximal grip force (Max_F_G_) exerted by all task-involved digits is shown for each grip type (5D, 3D_ane, 3D_mix), session (Anesthesia, Control), and subject groups (TIM and TRL group in **(A,B)** respectively). Data are mean values averaged across subjects. Error bars represent standard error of the mean. Asterisk indicates significant differences between sessions (*P* < 0.05).

The averaged Max_F_G_ (Mean ± SEM) across subjects under anesthesia and control sessions were plotted in Figure 2, for TIM (Figure 2A) and TRL (Figure 2B) groups separately. In MVC tasks, subjects exhibited significantly lower Max_F_G_ in the anesthesia session regardless of the grip type (main effect of *Session*: *F*_(1,16)_ = 47.91, *P* < 0.001). This observation is true for both groups of subjects no matter if subjects received anesthesia at TIM or TRL digits (no main effect of *Group*). Compared with the control session, maximal grip force was reduced by 48% in 5D, 44% in 3D_ane and 26% in 3D_mix for TIM group, 35% in 5D, 23% in 3D_ane, and 21% in 3D_mix for TRL group. In general, subjects were able to exert larger Max_F_G_ when using all the digits (5D) than three digits (3D_ane and 3D_mix) in the task (main effect of *Grip type*: *F*_(2,32)_ = 34.29, *P* < 0.001). However, exceptions were found in the anesthesia session showing similar Max_F_G_ between 5D and 3D_ane in TIM group, and similar Max_F_G_ between 5D and 3D_mix in TRL group (interaction effect of *Session × Grip type × Group*: *F*_(2,32)_ = 8.39, *P* < 0.001). Furthermore, Max_F_G_ produced by the three selected digits (3D_ane) was larger compared with that by the other three-digit combination (3D_mix) before anesthesia in the control session, but turned out to be smaller in the anesthesia session (interaction effect of *Session × Grip type*: *F*_(2,32)_ = 28.77, *P* < 0.001). The different Max_F_G_ observed in two 3D grip conditions was also varied in two subject groups (interaction effect of *Grip type × Group*: *F*_(2,32)_ = 21.98, *P* < 0.001). *Post hoc* tests revealed a larger Max_F_G_ for TIM group, yet a smaller Max_F_G_ for TRL group in 3D_ane compared with 3D_mix grip condition.

**Figure 3:**
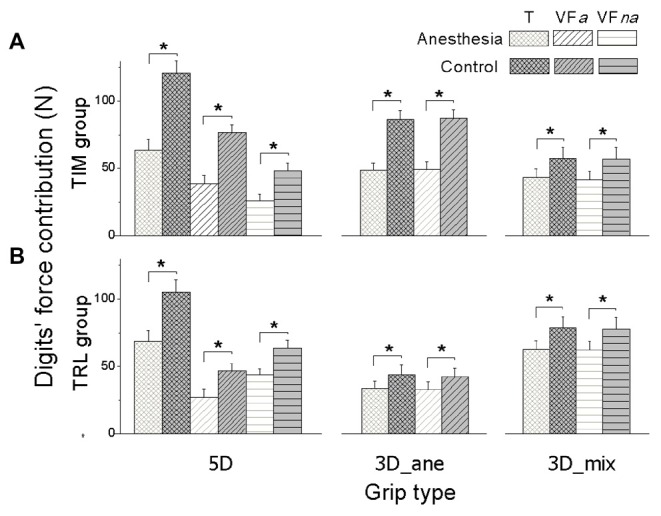
**Digits’ force contribution in MVC task.** Normal force contribution from the thumb (T), anesthetized virtual fingers (VFa) and non-anesthetized virtual fingers (VFna) when reaching the maximal grip force is shown for each grip type (5D, 3D_ane, 3D_mix), session (Anesthesia, Control), and subject groups (TIM and TRL group in **(A,B)** respectively). Data are mean values averaged across subjects. Error bars represent standard error of the mean. Asterisk indicates significant differences between sessions (*P* < 0.05).

In order to identify whether the overall Max_F_G_ decrease presented in anesthesia session was introduced by the anesthetized digits or not, we plotted the averaged Fn contribution in MVC tasks from the thumb (T), VF*a* and VF*na* across subjects in anesthesia and control sessions for each grip condition (Figure 3). In fact, all the digits exerted significantly less force to reach the Max_F_G_ after the anesthesia injection, including VF*na*, for which the Fn production dropped by 20.91 ± 0.9 N in 5D and 15.4 ± 1.61 N in 3D_mix grip conditions from control to anesthesia session (main effect of *Session*: *F*_(1,16)_ = 52.64, 59.51, and 12.36 in 5D, 3D_ane and 3D_mix, respectively; *P* < 0.005). In addition, digit units contributed to the Max_F_G_ differently. Specifically in 5D, thumb contributed the most when compared to both VF*a* and VF*na* in each session (main effect of *Digit units*: *F*_(2,32)_ = 124.09, *P* < 0.001) while VF*a* exerted larger Fn in the control session but less Fn in the anesthesia session than VF*na* (interaction effect of *Session* × *Digit* *units*: *F*_(2,32)_ = 14.43, *P* < 0.001). In addition, subjects in TIM group contributed more Fn from VF*a* than VF*na*, whereas subjects in TRL group presented the opposite (*Digit units* × *Group*: *F*_(2,16)_ = 17.48, *P* < 0.001). No significant difference was observed between the two subject groups (no main effect of *Group*), except in 3D_ane grip condition, subjects in TIM group produced larger force than TRL group during the control session (*Session × Group* interaction: *F*_(1,16)_ = 20.40, *P* < 0.001).

### OL Task Performance

Note that trial 1 was omitted, given the significant difference between the first and the subsequent trials. Thus, the results reported in OL task were based on averages across trial 2–28 (see data analysis). In OL task, subjects were asked to lift the object and keep it as vertical as possible. To evaluate subjects’ performance of object grasping and lifting, grip force and peak object roll were quantified in the following. Given no significant difference found between the two subject groups (TIM vs. TRL) for all the experimental variables regardless the grip types (no main or interaction effect of *Group*), data plotted in the following were therefore averaged over two groups for simplicity.

**Figure 4:**
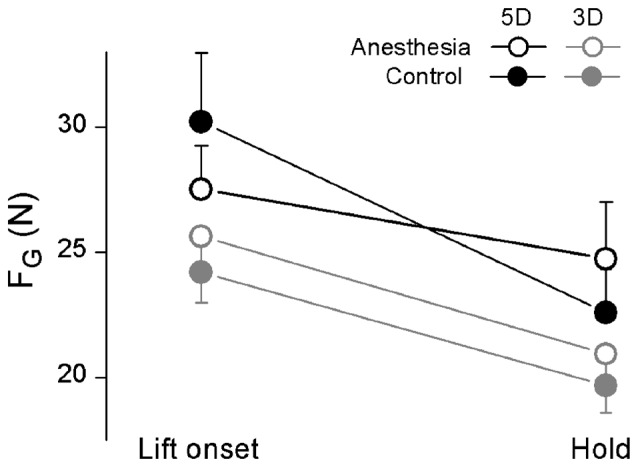
**Grip force at object lift onset and during object hold in OL task.** The grip force (F_G_) measured at lift onset (left) and during hold (right) is shown each grip type (5D and 3D) and session (Anesthesia, Control). Data are mean values averaged across trials two through 28 and further across subjects in both TIM and TRL groups. Error bars represent standard error of the across-subject mean.

Figure 4 showed the averaged F_G_ at object lift onset as well as during object hold across subjects in anesthesia and control sessions by using all five digits (5D) or three anesthetized digits (3D). In general, subjects dropped F_G_ production from object lift onset to object hold. However, such grip force decrease (ΔF_*G*_) in anesthesia and control sessions were dependent on the grip types (interaction *Session × Grip type*: *F*_(1,17)_ = 14.48, *P* < 0.05). Specifically, similar ΔF_G_ was observed in both anesthesia (4.70 ± 0.73 N) and control (4.53 ± 0.81 N) sessions by using 3D grip, whereas subjects showed significantly less ΔF_G_ in anesthesia (2.77 ± 0.80 N) than in the control session (7.64 ± 1.53 N) by using 5D grip (*P* < 0.05).

**Figure 5:**
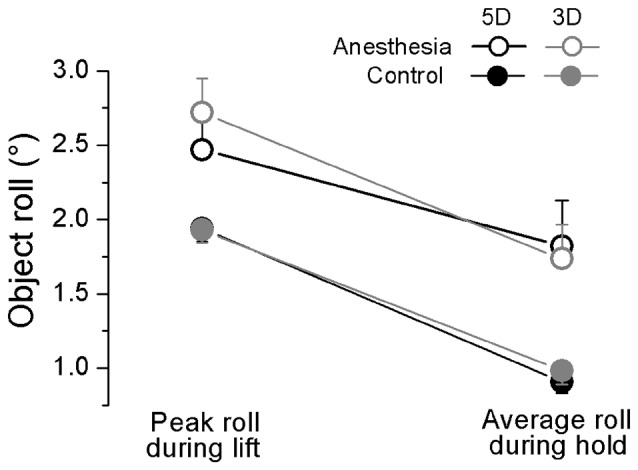
**Object roll during object lift and hold in OL task.** The absolute peak object roll measured during object lift and the averaged object roll during object hold phase is shown for each grip type (5D and 3D) and session (Anesthesia, Control). Data are mean values averaged across trials two through 28 and further across subjects in both TIM and TRL groups. Error bars represent standard error of the across-subject mean.

We plotted in Figure 5 the maximal object roll during OL and averaged object roll over hold phase for 5D and 3D grip conditions, averaged across subjects in anesthesia and control sessions. We observed a general larger object roll in the anesthesia (during lifting: 2.56 ± 0.22°; during hold: 1.78 ± 0.24°) than in the control session (during lifting: 1.93 ± 0.08°; during hold: 0.95 ± 0.08°) at both time epochs (main effect of *Session*: *F*_(1,17)_ = 10.56, *P* < 0.01), except during hold using 5D (*Session × Grip type × Phase* interaction: *F*_(1,17)_ = 6.81, *P* < 0.05). Additionally, all subjects generated significantly larger object roll during lift than hold phase (effect of *Phase*: *F*_(1,17)_ = 138.46, *P* < 0.001).

### Multi-Digit Coordination in OL Task

**Figure 6:**
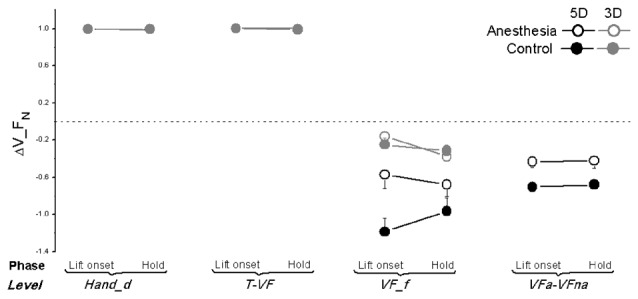
**Normal force synergy indices in OL task.** The synergy index quantifying multi-digit coordination of normal force output (ΔV_F_N_) is calculated at object lift onset and during object hold and shown for each grip types, session and hierarchical level (*Hand_d*: individual digits of the hand, *T-VF*, thumb vs. virtual finger; *VF_f*, individual fingers of the virtual finger; *VFa-VFna*, anesthetized vs. non- anesthetized virtual fingers). A dotted line denotes a zero line to distinguish positive (synergy presence) and negative (no synergy presence) ΔV indices. Data are mean values averaged across subjects in both TIM and TRL groups. Error bars represent standard error of the across-subject mean.

**Figure 7:**
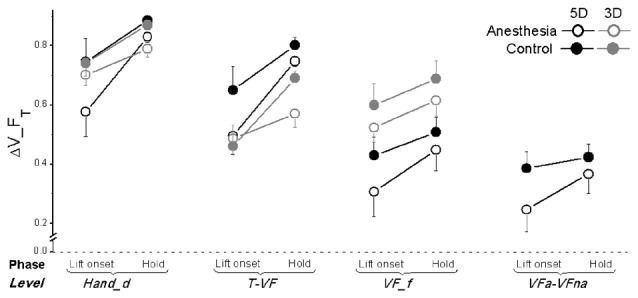
**Tangential force synergy indices in OL task.** The synergy index quantifying multi-digit coordination of tangential force output (ΔV_F_T_) is calculated at object lift onset and during object hold and shown for each grip types, session and hierarchical level (*Hand_d*: individual digits of the hand, *T-VF*, thumb vs. virtual finger; *VF_f*, individual fingers of the virtual finger; *VFa-VFna*, anesthetized vs. non- anesthetized virtual fingers). A dotted line denotes a zero line to distinguish positive (synergy presence) and negative (no synergy presence) ΔV indices. Data are mean values averaged across subjects in both TIM and TRL groups. Error bars represent standard error of the across-subject mean.

**Figure 8:**
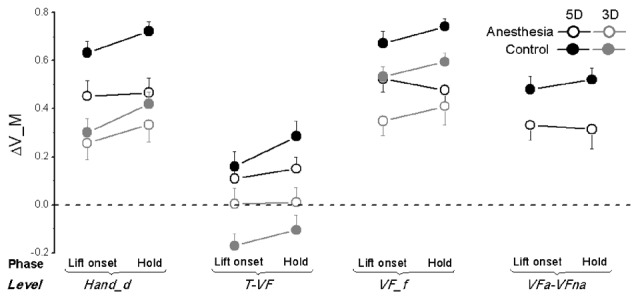
**Moment of force synergy indices in OL task.** The synergy index quantifying multi-digit coordination of moment of force output (ΔV_M) is calculated at object lift onset and during object hold and shown for each grip types, session, and hierarchical level (Hand_d: individual digits of the hand, T-VF, thumb vs. virtual finger; VF_f, individual fingers of the virtual finger; VFa-VFna, anesthetized vs. non- anesthetized virtual fingers). A dotted line denotes a zero line to distinguish positive (synergy presence) and negative (no synergy presence) ΔV indices. Data are mean values averaged across subjects in both TIM and TRL groups. Error bars represent standard error of the across-subject mean.

As previously stated, the synergy indices stabilizing the normal force, tangential force as well as the moment of force in the object frontal plane have been calculated at four different hierarchical levels: *Hand_d, T-VF, VF_f* and *VFa-VFna*. We plotted ΔV_F_N_, ΔV_F_T_, and ΔV_M averaged across subjects within the anesthesia and control sessions at object lift onset and hold in Figures 6–8 respectively. As shown in Figure 6, different ΔV_F_N_ values were observed at four hierarchical levels (main effect of *Hierarchical level*: *F*_(1.08,17.32)_ = 375.16 in 5D; *F*_(1.00,16.00)_ = 1341.0 in 3D; both *P* < 0.001). Specifically, subjects presented high values of ΔV_F_N_ (+1) at both levels involving the thumb (*Hand_d* and *T-VF*) regardless of the sessions, phases or grip types. In contrast, all subjects showed negative values of ΔV_F_N_ at levels involving only VF fingers (*VF_f* and *VFa-VFna*): *t-value* within the range of (−9.676; −2.260) across individual comparisons; all *P* < 0.05. Given the absence of synergy, ΔV_F_N_ was not investigated further.

As plotted in Figure 7, ΔV_F_T_ was smaller by 0.1 in the anesthesia compared with the control session, even though the observed discrepancy between the sessions was significant only at the level of *Hand_d* (main effect of *Session*: *F*_(1,17)_ = 14.23; *P* < 0.05) and T-VF (main effect of *Session*: *F*_(1,17)_ = 6.02; *P* < 0.05). In addition, significantly lower ΔV_F_T_ was observed at object lift onset than object hold (main effect of *Phase*: *F*_(1,17)_ = 31.23 for *Hand_d*, *F*_(1,17)_ = 33.40 for T-VF, *F*_(1,17)_ = 7.47 for *VF_f*; all *P* < 0.05), except at *VFa-VFna* level. Moreover, subjects altered their synergy index of tangential force when using different grip types: at the level of T-VF, a significantly higher ΔV_F_T_ was observed in 5D than 3D grip (main effect of *Grip type*: *F*_(1,17)_ = 1.81, *P* < 0.001); at the level of *VF_f*, however, ΔV_F_T_ was significantly lower in 5D than 3D grip (main effect of *Grip type*: *F*_(1,17)_ = 17.69, *P* < 0.005). Furthermore, ΔV_F_T_ showed varied values at different hierarchical levels (main effect of *Hierarchical level*: *F*_(1.3,20.80)_ = 53.14 in 5D; *F*_(1.04,16.58)_ = 20.26 in 3D; both *P* < 0.001). For both grip types, subjects presented the highest ΔV_F_T_ at the level of *Hand_d*. Additionally in 5D grip, the lowest ΔV_F_T_ was observed at the level of *VFa-VFna*.

Similar to ΔV_F_T_, subjects also exhibited lower values of ΔV_M after digital anesthesia (0.32 ± 0.05) compared with controls (0.43 ± 0.08) as shown in Figure 8. This observation is significant for level of *VF_f* and *VFa-VFna* (main effect of *Session*: *F*_(1,17)_ = 17.38 and 10.47, respectively; both *P* < 0.01) as well as for level of *Hand_d* but only for 5D grip condition (interaction effect of *Session × Grip type*: *F*_(1,17)_ = 6.66, *P* < 0.05). Not surprisingly, subjects increased their synergy index of ΔV_M during hold from object lift onset, which were significant for levels of *Hand_d* and T-VF (main effect of *Phase*: *F*_(1,17)_ = 8.31 and 6.10, respectively; both *P* < 0.05). In addition, we also observed varied ΔV_M by using different grip types. Particularly, subjects showed significantly higher values in 5D than 3D at individual digit levels, i.e., *Hand_d* and *VF_f* (main effect of *Grip type*: *F*_(1,17)_ = 23.77 and 12.37, respectively; both *P* < 0.005), as well as at T-VF level, but in the control session only (interaction *Session × Grip type*: *F*_(1,17)_ = 4.95, *P* < 0.05). Moreover, subjects presented different ΔV_M calculated at different hierarchical levels for both 5D and 3D grip types (main effect of *Hierarchical Levels*: *F*_(1.22,19.56)_ = 74.12 in 5D, *F*_(1.21,19.29)_ = 90.31 in 3D; both *P* < 0.001). We observed ΔV_M values at T-VF level were the smallest of all levels regardless of grip types, and furthermore, for 3D grip type ΔV_M was significantly larger at *VF_f* level than at *Hand_d* level for subjects in the control session only (interaction* Session × Level*: *F*_(2,32)_ = 11.20, *P* < 0.05).

### Martini Glass Task

**Figure 9:**
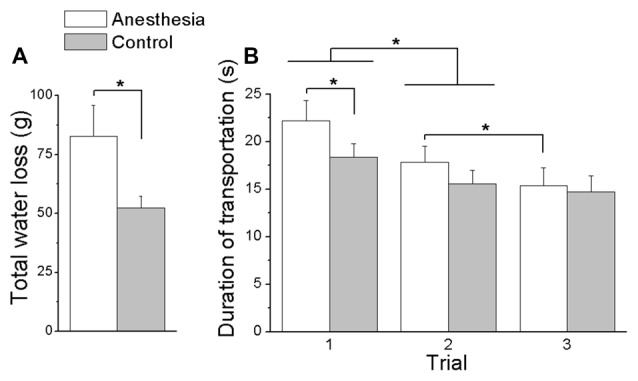
**Total water loss and duration of transportation in Martini glass task. (A)** Shows the overall water spilled cumulated over three trials in the Martini glass task for both anesthesia and control sessions. **(B)** Shows the duration of transportation in the Martini glass task for each trial and session. Data are mean values averaged across subjects in both TIM and TRL groups. Error bars represent standard error of the across-subject mean. Asterisk indicates significant differences between sessions (*P* < 0.05).

For the martini glass task, we plotted the averaged subjects’ martini glass task performance, i.e., total water loss and duration of transportation, for both the anesthesia and control sessions in Figure 9. As seen from Figure 9A, subjects spilled more water in the anesthesia (82.89 ± 12.96 g) compared with the control session (52.28 ± 4.97 g) regardless of the group (main effect of *Session*: *F*_(1,16)_ = 6.44, *P* < 0.05). Figure 9B shows that, during the first trial, subjects in the anesthesia session spent more time to transport the martini glass filled with water between two instructed locations (anesthesia: 22.19 ± 2.15 s; control: 18.36 ± 1.40 s). However, in subsequent trials (trial 2 and trial 3), subjects throughout both sessions completed the task with similar duration of glass transportation (interaction *Session × Trial*: *F*_(2,32)_ = 3.76, *P* < 0.05). Subjects in both sessions showed a decrease of transportation duration from the first (20.3 ± 1.7 s) to the second trial (16.7 ± 1.4 s; main effect of *Trial*: *F*_(2,32)_ = 23.86, *P* < 0.001). No further decrease was observed from the second (15.6 ± 1.5 s) to the third trial (14.8 ± 1.7 s) in the control session, however, subjects in the anesthesia session continued with shortening time in the subsequent trials (Trial 2: 17.8 ± 1.7 s; Trial 3: 15.4 ± 1.9 s; *P* < 0.005). No main or interaction effect of *Group* was found in these martini glass task performance variables.

## Discussion

### Effect of Local Anesthesia on Digital Maximal Grip Force Abilities

In MVC tasks, subjects showed decreased maximal force application in grasping after digital anesthesia injections (Figure 2). This result is consistent with previous reports in pinch conditions (Augurelle et al., 2003) as well as in individual or four-finger isometric pressing tasks (Shim et al., 2012; Kim et al., 2013). These findings indicate that the deficiency of sensory feedback would reduce the MVC ability in the digits. The force reduction induced by local anesthesia, may be due to decreased cortical motoneuron excitability after sensory flow is altered. For example, Rossini et al. (1996) found that anesthesia of the median and radial nerves induced a decrement of MEPs amplitude in first dorsal interosseous muscle. Nevertheless, experimental sets up for earlier findings have been limited to the local (anesthetized) digits, thus overlooked the interactive adaptations among local and non-local (non-anesthetized) digits. Our current results revealed that the deficit in maximal force production presented not only on deafferent digits but also on intact digits.

First, the significant drop in Max_F_G_ appeared in all three grip types, i.e., anesthetized thumb opposing two anesthetized and two non-anesthetized fingers, anesthetized thumb opposing two anesthetized fingers, and anesthetized thumb opposing two non-anesthetized fingers. Since the thumb is the only digit on its side in the grasp, opposing the other fingers, it mechanically contributes 50% to the overall grip force. Therefore, given thumb is the only digit systematically anesthetized throughout the grip types, the thumb force deficit (Li et al., 2004) could constrain the overall Max_F_G_ amplitude, leading to a general grip force drop. However, this is true only when the thumb is physically weaker than its opposing fingers, such as in 5D. The removal of somatosensory feedback from three digits resulted in similar Max_F_G_ regardless if subjects used all five digits or three anesthetized digits (i.e., T, I and M). That is, adding two more fingers (R and L) in the grip didn’t raise the overall grip force in the anesthesia session as it did in the controls, despite the potential intact sensation input from the added fingers (Figure 2, TIM group). Despite this, the post-anesthesia thumb-constrained Max_F_G_ was not applied in all the grip types, since the amplitude of Max_F_G_ was not consistent across grip types in anesthesia session (Figure 2). The lowest Max_F_G_ and thus the thumb Fn was produced by enrolling thumb, ring and little fingers in the MVC task (i.e., 3D_mix in TIM group, 3D_ane in TRL group). In these conditions, Max_F_G_ was most likely constrained by the VF Fn production (in R and L) due to its weaker absolute strength compared to the thumb. These observations lead to a very interesting finding, in that, the digital anesthesia in local digits also affected the MVC in the non-local digits, inducing the maximal grip force disability.

Further results in digits’ force contribution showed a universal Fn drops in the anesthesia session from all the task-involved digits, including the non-anesthetized digits in 5D (Figure 3). These novel findings showed that the effect of digital anesthesia on the maximal ability in grip force production is grip type-dependent based upon local and non-local fingers involvement. The absent somatosensory feedback induced by selective digital anesthesia led to impairment in the MVC at both local and non-local digits, resulting in subjects’ reduced ability in maximal grip force production. Local and non-local responses have been examined in some earlier studies during submaximal grip force tasks in healthy hands. Aoki et al. (2007) investigated the role of tactile information in adapting grip object properties, and found that changes in texture at a given digit elicit force adjustments at the same as well as other digits, indicating that sensory information at one digit affects the force modulation at non-stimulated digits. Our current results further implied that individual sensory input is shared across all the digits and the disturbed signal from local sensory channel(s) has a more comprehensive impact on the process of the motor output execution in the sensorimotor integration.

### Absent Digital Sensation Induced Deficits in Force and Moment Control

In object lift task, the subjects didn’t present observable erroneous actions such as dropping the device after selective digital anesthesia. In particular, subjects were able to exert sufficient F_G_, regardless the involved digits were all anesthetized (3D) or selectively anesthetized (5D). Furthermore, subjects presented relative similar F_G_ before and after digital anesthesia at both lift onset and during hold (Figure 4). These observations suggest that capabilities in grip force control can be preserved even if the somatosensory information is disturbed. This may be because the residual sensory input available in the hand and/or forearm was recruited to assist the motor output in the sensorimotor integration process. Note that our results didn’t show an excessively large F_G_ as reported in earlier studies after nerve block at either digital level (Johansson and Westling, 1984; Monzée et al., 2003) or lower median nerve level (Dun et al., 2007). The discrepancy between the current and previous results might due to the different experimental designs. First, previous studies have been limited to pinch grip with both involved digits anesthetized. Second, the objects used in their lift task were much lighter (varied from 50 g to 400 g) than in the current study (665 g). Three-digit grasping has been explored by Nowak et al. (2001), but during a dynamic oscillation not steady hold task. We suspect that the sensory information originated from skin deformation could enhance the residual proprioception (Edin and Johansson, 1995) in the hand and/or the forearm with increased cutaneous strain when lifting a much heavier object. Recent studies have investigated the effect of digital sensory deficit introduced by CTS on grasp control. They found a remarkable larger grip force compared with healthy controls in 5D involving both affected and non-affected digits, but not in 2D or 3D when all involved digits were affected (Zhang et al., 2011, 2012, 2013). Such grip force increase in CTS patients during the whole hand grasp might be a strategic adaptation in force control as a result from erroneous actions in daily life activities, such as frequent object drops. However, the digital anesthesia applied in our study artificially removed the tactile and proprioception input from the digits in a temporary fashion. In contrast to the patients who suffered tactile sensory impairment gradually and chronically, our subjects had no explicit pre-knowledge regarding their sensory deficiency and consequential motor output.

Despite the residual ability of grip force control discussed above, our results also revealed subtle behavioral inefficiencies in the whole hand grasp while digital sensory inputs were selectively blocked. Grip force is exerted at the time of lift onset, following anticipatory control fashion (Fu et al., 2010). Therefore, the general decrease in F_G_ from lift onset to the object holding phase implies a correction from an overshoot output based on feedforward control mechanism. This correction results in a more efficient force pattern (i.e., closer to task-required minimum force) when online feedback information is utilized to determine the behavioral performance (e.g., object roll), such as, visual or residual somatosensory sensory input from the hand and/or forearm. In this scenario, the F_G_ drop between two time epochs reflects an ability of sensorimotor adaptation responsible to object property (i.e., weight). Our results showed that subjects maintained the within-trial force adaptation ability in 3D but not in 5D after three digits’ anesthesia (Figure 4). This is an interesting yet counterintuitive finding, since utilizing all anesthetized digits lead to “normal” force adaptation, but recruiting non-anesthetized digits with intact sensory output resulted in a deficit of force control. This result prompted a central processing mechanism in the sensorimotor integration, that is, the accurate input available from intact sensory channels is not prioritized over the disturbed sensory input when both coexist. Instead, the central controller will integrate all the sensory signals with varied precision, and utilize the processed information for updating the task-relevant motor program execution. This assumption imposes a larger challenge to CNS in a motor task when recruiting mixed sensory signals with unequally disturbed perception compared to a uniformly impaired sensory system. This assumption is in accordance with our previous study on CTS patients, which demonstrated an inefficient grip force production when adding intact digits in the grip, but not for sensory-impaired digits only (Zhang et al., 2013).

In addition, subjects presented with a decreased ability to maintain the object’s vertical orientation during lift or transportation. Our results showed that removing the cutaneous and proprioception sensory information led to a larger object roll during object lift as a result of anticipatory control and object hold following the online motor adaptation control (Figure 5). Similar abated performance was been observed in 3D and 5D, indicating the intact sensory channels available from non-anesthetized digits in 5D were not capable to help to improve the task performance that was induced by impaired sensory channels. Correspondingly, subjects’ deficit in moment control was also reflected in the daily life task (i.e., martini glass transportation), as evidenced by the larger amount of water spill and slower transportation speed after digital anesthesia (Figure 9). These results manifest the difficulty in moment control elicited by sensory deficiency in grasping manipulative tasks. In studies when resultant moment was not required to be maintained (e.g., no need to keep the object vertical orientation), or when the feedback on object roll induced by erroneous moment production was not provided (e.g., object was physically constrained and allows no tilt), an inefficient moment production was observed in a pinch grip after digital anesthesia (Monzée et al., 2003). In our current study, the object tilting and water spilling could be evaluated as the erroneous output based on visual and/or residual sensory feedback from the hand and the forearm. In this scenario, subjects’ failure of accurate moment production indicates that residual sensory information is not sufficient to assist the motor correction in moment control process.

### Feedback Control Component Resides in Motor Synergy Structure

Very few studies have determined the multi-digit coordination patterns underneath digital anesthesia conditions. Koh et al. (2015) investigated motor synergies based on the force across-trial variability among four fingers in isometric pressing tasks. As a result, there was no synergy drop identified in the absence of digital sensory feedback. The reported preserved synergy might due to the dominant role of visual feedback in accurate force production and correction compared with cutaneous sensory modality (Hartcher-O’Brien et al., 2008; Touzalin-Chretien et al., 2010). Our current study examined the effect of digital anesthesia on multi-digit coordination in object grasp and lift control. Most importantly, we quantified the multi-digit synergy index (reviewed in Latash et al., 2003) responsible for task-specific force and moment control at different hierarchy levels involving digits with and without sensory deficiency. One general result is that multi-digit motor synergies were presented (i.e., ΔV > 0) in stabilizing the total or subtotal Fn (Figure 6), F_T_ (Figure 7) or the moment in frontal plane (Figure 8), yet to different extents and at different levels. Even though the nearly perfect synergy index value (close to 1) was observed for Fn, indicating a high covariation structure across the individual force output among digits (*Hand_d*) as well as between thumb and virtual finger (*T-VF*), it was not given much attention. This is because the “perfect” coordination of Fn by the thumb and fingers was artificially formulated by the task mechanics, i.e., the Fn produced by the thumb should be equal but opposite to that exerted by VF in object lift task. However, when the force coordination was not mandated among the digits within VF side (i.e., at the level of *VF_f* and *VFa-VFna*), synergy disappeared in both control and anesthesia sessions. This suggests that the subtotal normal force from the involving fingers is not intentionally stabilized and controlled from the central level in hand grasp control task.

Different from normal force, we found synergistically motor coordination patterns for both tangential force or moment outputs across the task-involved digits. Grasp task has been viewed from two-subtask perspectives: object lift and its orientation maintenance. The parallel presence of F_T_ synergy and moment synergy implies a superposition principle of force control and moment control in object grasping task (Zatsiorsky et al., 2004). The general observed positive values of ΔV_F_T_ and ΔV_M at different levels imply a covariation structure among the elemental variables, revealed as the mechanical outputs from different hierarchical control levels involving either the subsets of digits (e.g., T-VF and VFa-VFna) or the individual digits (e.g., Hand_d and VF_f). The observed higher ΔV indices at levels of Hand_d and VF_f for both synergies complies with an earlier theoretical assumption, which is, synergies co-exist and are lack of interference (Zhang et al., 2008). This finding further indicates a parallel but independent process of tangential force and moment stabilization, which was mostly achieved by covariation at the individual digits level. Particularly, the overall F_T_ was stabilized primarily by coordinating Ft across individual digits including the thumb (i.e., highest ΔV_F_T_ at level of Hand_d). The resultant moment, however, was stabilized mainly by coordinating the moment from individual fingers at the VF side (i.e., highest ΔV_M at level of VF_f). The discrepancy of effector involvement between the synergies may relate to the different role of the thumb in force vs. moment control. The former controller requires upward Ft production from the thumb as well as from the other digits, whereas the latter controller may view the thumb as the pivot point for moment production by other fingers.

Most importantly, the presented force or moment synergies were affected and weakened by selective digital sensory blocks. The attenuated motor synergies present underneath digital anesthesia reveals a feedback control component in formation process for the motor output covariation structure. Consequently, a defective moment coordination pattern induced by digital anesthesia was embodied in notable behavioral features, such as object roll. The role of sensation feedback in motor synergy structure has been discussed controversially in different schemes or models. For example, feedback signals have been proposed to assist coordinating output signals of finger forces in optimal control schemes (Todorov and Jordan, 2002) and in a model based on action of central back-coupling loops (Latash et al., 2005). Contrarily, a feed-forward scheme suggested that the formation of motor variabilities structure may not consist any explicit feedback correction mechanisms (Goodman and Latash, 2006). The problem is that these computational models need to be testified in experimental protocols that allow eliminating or altering sensory feedback input, different digits’ enrollment, and accurate moment control. Our study emphasized that a sensory deficit at a subset of digits impaired the motor coordination patterns among all involved digits. The influential effect of digital anesthesia on task-specific motor synergies corroborated the favorable mechanism of feedback-based coordination of the elemental motor variables, indicating a peripheral sensory deficit induced changes in the CNS control mechanism. This hypothetical control mechanism needs further investigations in more moment control tasks.

## Conclusion

In summary, our results suggested that the absence of somatosensory information induced motor deficits in hand grasp control, as evidenced by reduced maximal force production ability in both local and non-local digits, impairment of force and moment control in object lift and hold, and attenuated motor synergies in stabilizing task performance variables, namely the tangential force and moment of force. These findings implied that individual sensory input is shared across all the digits and the disturbed signal from local sensory channel(s) has a more comprehensive impact on the process of the motor output execution in the sensorimotor integration process. Additionally, a feedback control mechanism with a sensory-based component resides in the formation process for the motor covariation structure.

## Author Contributions

WZ contributed to the design of the study. AC, WZ, KM, CG, ER and MT contributed to subject recruitment and data acquisition. BH and CB contributed to subject screening, conducted digital nerve block procedure and assisted with data acquisition. AC and WZ performed data analyses, data interpretation and manuscript preparation. All authors contributed to manuscript revision, and approved the final manuscript.

## Funding

This project was in part supported by PSC-CUNY Awards (68854-0046), and Doctoral Student Research Grant from Graduate Center of the City University of New York.

## Conflict of Interest Statement

The authors declare that the research was conducted in the absence of any commercial or financial relationships that could be construed as a potential conflict of interest.

## Footnotes

^1^Sensory neuron fibers are generally more sensitive to digital nerve block than motor neuron fibers according to a characteristic of local anesthetic actions: nerves with higher firing frequency and more positive membrane potential are more sensitive to local anesthetic block. Sensory fibers, especially pain fibers, have a high firing rate and relatively longer action potential duration than motor fibers, and thus are more sensitive to lower concentrations of local anesthetics.
